# First-Principles Study of the Consequences of Cerium Doping on the Photocatalytic Activity of Zinc Oxide

**DOI:** 10.3390/ijms252313033

**Published:** 2024-12-04

**Authors:** Eimy Y. Rodriguez-Mena, Luis A. Alcalá-Varilla, José D. Ortiz-Romero

**Affiliations:** 1 Departamento de Física y Electrónica, Universidad de Córdoba, Montería 230002, Córdoba, Colombia; erodriguezmena39@correo.unicordoba.edu.co (E.Y.R.-M.); jortizromero@correo.unicordoba.edu.co (J.D.O.-R.); 2 Programa de Ingeniería de Sistemas, Universidad Cooperativa de Colombia, Montería 230002, Córdoba, Colombia

**Keywords:** photocatalytic activity, ZnO, doped cerium, DFT, fermi level

## Abstract

Recent experimental studies have shown that the photocatalytic activity of zinc oxide is enhanced when doped with cerium and that these enhancements depend on the doping concentration; in particular, the highest photocatalytic activity rates have been reported for cerium concentrations in zinc oxide close to 3.00% or 5.00%. So far, there is no sufficient explanation why the maximum photocatalytic activity rates of cerium-doped zinc oxide occur for the above concentrations. The main objective of this work is to try explain the above-mentioned. For this purpose, we performed a study based on density functional theory on the effects generated on the structural and electronic properties of different cerium concentrations in zinc oxide, and found that the relative position of the fermi level (closeness to the valence band) could be related to the peaks of a higher photocatalytic activity of $Zn_{1-x}Ce_{x}O$. We also found that for a low cerium concentration rate, the value of the *c* lattice parameter became lower than the value it had in pure ZnO, while the value of the *a* lattice parameter of the $Zn_{1-x}Ce_{x}O$ system was always higher than the value observed in pure ZnO.

## 1. Introduction

The Earth’s atmosphere is altered mainly by human activity, leading to global warming. This phenomenon is linked to the greenhouse effect which has intensified during the past six decades [1]. The greenhouse effect involves the amount of solar energy that reaches the Earth’s surface, the infrared radiation that the Earth reflects, and the greenhouse gases that retain this radiation. Carbon dioxide ($CO_{2}$) is the primary greenhouse gas and much of the anthropogenic emissions of this gas are caused by the energy generation mechanism using fossil fuels which has a major impact on both air quality degradation and global warming [2,3,4]. Photocatalysis is a process that emulates natural photosynthesis and presents a viable solution for mitigating $CO_{2}$ effects by utilizing solar energy to initiate chemical reactions that convert those toxic gasses into useful chemicals and fuels under ambient temperature and air pressure [5].

Among the most studied and used photocatalytic materials is titanium dioxide ($TiO_{2}$) [6,7,8,9,10]; however, some studies have reported that zinc oxide (ZnO) presents a higher photocatalytic activity than that exhibited by ($TiO_{2}$) in the decolorization of some dyes [11,12]. Moreover, ZnO has many advantages, such as its manufacturing simplicity, chemical stability, high electron mobility, and high efficiency in absorbing ultraviolet radiation [13,14]; for these reasons, studies on photocatalysis using ZnO have increased in the last years [15,16,17,18,19].

Despite its excellent attributes, ZnO has problems due to its large band gap (3.37 eV) and the fast recombination rate of the photogenerated electron–hole pair, which reduces its photocatalytic activity. One way to counteract these limitations is to introduce impurities on the material, for example, ZnO has been doped with Mg, Al, Cu, and Ni, among others elements [20,21,22,23]

In particular, doping ZnO with rare earth atoms has attracted attention. According to Jin-Chung et al. [24] the addition of rare earth ions can generate midgap states in the energy band gap. Among the rare earth atoms, cerium is the most prevalent in nature and has gained much attention due to its special properties. As noted by Bechambi et al. [25], cerium easily forms oxygen vacancies and acts as an electron reservoir, increases the electron–hole pair recombination time. As a consequence, there are several theoretical and experimental studies about cerium-doped ZnO [26,27,28,29,30,31,32]. In particular, M. Ahmad et al. [26] carried out experimental studies on the behavior of the photocatalytic activity regarding the cerium concentration (from 0.50% to 10.00%), and found that the higher photocatalytic activity occured at a value near 3.00%. Meanwhile, another experimental study by Pathak et al. [27] showed that the peak of the photocatalytic activity was produced near a cerium concentration of 5.00%.

To the best of our knowledge, there is currently no clear explanation for why the highest rates of photocatalytic activity of $Zn_{1-x}Ce_{x}O$ occur at concentrations close to 3.00% or 5.00%. Although there are theoretical studies on the properties of the $Zn_{1-x}Ce_{x}O$ system, such as those carried out by Yue Feng et al. [31] and X.F. Jia et al. [32], they do not present a complete explanation of why the maximum peaks for photocatalytic activity occur for concentrations close to 3.00% or 5.00%, as these works only use a range of concentrations higher than 3.00%. This motivated us to study this system through a theoretical approach based on density functional theory in order to provide an explanation for why the highest photocatalytic activity of the $Zn_{1-x}Ce_{x}O$ occurs with concentrations close to 3.00% or 5.00%. For this purpose, in the present work, concentrations lower and higher than 3.00% were addressed (1.39–12.50%). The main result of this work showed that the relative position of the Fermi level could have an important role in explaining the higher photocatalytic activity observed for the $Zn_{1-x}Ce_{x}O$ system at a cerium concentration close to 3.00%.

## 2. Results and Discussion

### 2.1. Structural Properties

The structural properties of $Zn_{1-x}Ce_{x}O$ (where x= 0.0139, 0.0185, 0.0278, 0.0313, 0.0417, 0.0625, and 0.1250) were studied.

The bond lengths, angles, and lattice parameters found in this study and the lattice parameters of other experimental and theoretical works are reported in Table 1.

The *a* and *c* lattice parameters found for pure ZnO in this work were in agreement with those reported in experimental studies, such as those by Miha Ravbar et al. [33], Vijayaprasath et al. [29], and Parangusan et al. [30]; in addition, the lattice parameters found in this work for the systems $Zn_{0.9686}Ce_{0.0313}O$, $Zn_{0.9583}Ce_{0.0417}O$, and $Zn_{0.875}Ce_{0.125}O$ were close to the experimental values reported by Vijayaprasath et al. [29] (for 3.00% and 6.00%) and by Parangusan et al. [30] (from 3.00% to 12.00%). Our results are also in agreement with other theoretical studies carried out by Yue Feng et al. [31] and X.F Jia et al. [32]. It can be seen that the lattice parameters were near to the values obtained in the systems of $Zn_{0.9686}Ce_{0.0313}O$, $Zn_{0.9583}Ce_{0.0417}O$, and $Zn_{0.9375}Ce_{0.0625}O$ in this investigation. The above discussion allowed us to validate the methodology that was implemented in this study.

In addition, this paper presents information about the bond lengths and angles not reported in other experimental or theoretical studies for pure zinc oxide and cerium-doped zinc oxide for cerium concentrations from 1.39% to 12.50%. Data for cerium concentrations lower than 3.00% were also reported, which had not been previously reported in other theoretical studies and were important for the analysis of the behavior of the photocatalytic activity of the system.

**Table 1:** Bond lengths, angles, and lattice parameters for pure zinc oxide and cerium-doped zinc oxide from 1.39% to 12.50%, where $b_{1}$ represents the bond length Ce-*O* and $b_{2}$ represents the bond length Zn-*O*. α,θ, and β represent the angles formed by *O*-Ce-O, Zn-*O*-Zn, and Zn-*O*-Ce, respectively, as shown in Figure 1. Abbreviations: Exp: Experimental.

Reference	$b_{1}\left(Å\right)$	$b_{2}\left(Å\right)$	α(°)	θ(°)	β(°)	a(Å)	c(Å)
ZnO	-	1.991	-	108.662	-	3.267	5.273
$Zn_{0.9861}Ce_{0.0139}O$	2.267	1.997	108.203	113.323	105.080	3.329	5.268
$Zn_{0.9815}Ce_{0.0185}O$	2.270	1.999	108.168	113.274	105.136	3.290	5.273
$Zn_{0.9722}Ce_{0.0278}O$	2.275	2.001	108.117	113.195	110.790	3.297	5.284
$Zn_{0.9686}Ce_{0.0313}O$	2.268	1.976	111.109	114.590	107.785	3.294	5.305
$Zn_{0.9583}Ce_{0.0417}O$	2.269	1.978	110.540	114.379	103.322	3.302	5.317
$Zn_{0.9375}Ce_{0.0625}O$	2.275	1.984	110.402	114.143	103.702	3.319	5.339
$Zn_{0.875}Ce_{0.125}O$	2.342	2.004	109.054	110.987	104.773	3.373	5.394
Exp ZnO [33]	-	-	-	-	-	3.242	5.194
Exp ZnO [29]	-	-	-	-	-	3.254	5.211
Exp (3.0%) [29]	-	-	-	-	-	3.252	5.213
Exp (6.0%) [29]	-	-	-	-	-	3.253	5.215
Exp ZnO [30]	-	-	-	-	-	3.249	5.204
Exp (3.0%) [30]	-	-	-	-	-	3.252	5.209
Exp (6.0%) [30]	-	-	-	-	-	3.253	5.210
Exp (12.0%) [30]	-	-	-	-	-	3.255	5.214
ZnO [31]	-	-	-	-	-	3.242	5.211
$Zn_{0.9686}Ce_{0.0313}O$ [31]	-	-	-	-	-	3.297	5.275
$Zn_{0.9583}Ce_{0.0417}O$ [31]	-	-	-	-	-	3.301	5.314
$Zn_{0.9375}Ce_{0.0625}O$ [31]	-	-	-	-	-	3.328	5.365
ZnO [32]	-	-	-	-	-	3.289	5.308
$Zn_{0.9686}Ce_{0.0313}O$ [32]	-	-	-	-	-	3.315	5.344
$Zn_{0.9583}Ce_{0.0417}O$ [32]	-	-	-	-	-	3.328	5.346
$Zn_{0.9375}Ce_{0.0625}O$ [32]	-	-	-	-	-	3.338	5.364

**Figure 2:**
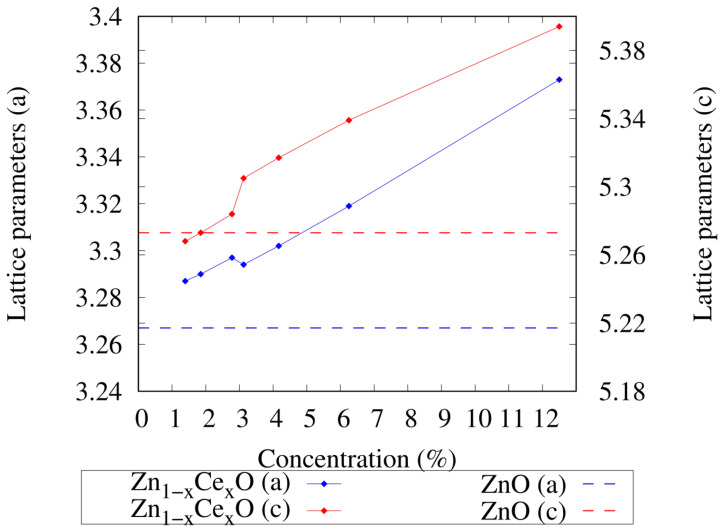
Study of the lattice parameters as a function of the cerium concentration of the system $Zn_{1-x}Ce_{x}O$. The blue line corresponds to the values of the *a* lattice parameter and the red line represents the values of the *c* lattice parameter. In both cases, the horizontal lines with dashes correspond to the lattice parameter values for the pure bulk of ZnO.

When comparing all cerium-doped zinc oxide systems with the pure zinc oxide system, changes in the lattice parameters were observed, i.e., the addition of cerium increased the lattice parameters. It can also be seen that as the cerium concentration decreased, the lattice parameters also decreased; this behavior was also reported by [31,32]. On the other hand, from this study, it can be seen that the lattice parameters at cerium concentrations around 3.00% showed abrupt changes when the cerium concentration was close to 3.00%, as shown in Figure 2. This could indicate that some phenomenon associated with the photocatalytic activity of $Zn_{1-x}Ce_{x}O$ could be occurring for concentrations close to 3.00%. From Figure 2, it can also be seen that for low concentrations (1.39%), the value of the *c* lattice parameter for the system $Zn_{1-x}Ce_{x}O$ became lower than in the pure zinc oxide ZnO, while the value of the *a* lattice parameter remained higher than the value in ZnO. It is important to highlight that the value of the c/a relation of the $Zn_{1-x}Ce_{x}O$ system closest to the value of this relation in the pure bulk of ZnO (c/a=1.614) was the one that corresponded to the concentration of 3.13% (c/a=1.611).

### 2.2. Electronic Structures

**Figure 3:**
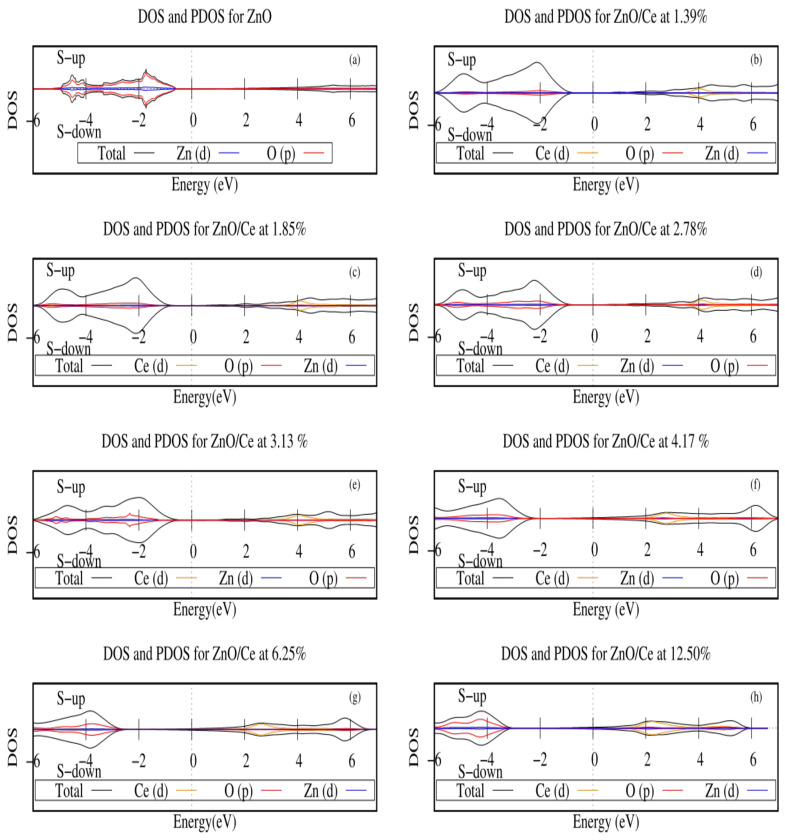
DOS and PDOS for (**a**) Pure zinc oxide; (**b**) Cerium-doped zinc oxide at 1.39%; (**c**) Cerium-doped zinc oxide at 1.85%; (**d**) Cerium-doped zinc oxide at 2.78% ; (**e**) Cerium-doped zinc oxide at 3.13%; (**f**) Cerium-doped zinc oxide at 4.17%; (**g**) Cerium-doped zinc oxide at 6.25%; (**h**) Cerium-doped zinc oxide at 12.50%. The Fermi level is set at zero energy.

In this section, we studied the electronic properties of cerium-doped zinc oxide. Figure 3 shows the density of states for the systems studied; here, the Fermi level was set at zero energy.

The density of states of pure ZnO and its projections are shown in Figure 3a, in which we can appreciate a semiconductor behavior, where it is found that the orbitals that contribute the most to DOS in the valency band (VB) and conduction band (CB) were the *p* orbital of oxygen and the *d* orbital of zinc, also there was no magnetization for this material.

Figure 3b–h shows the density of states and the projections for the $Zn_{1-x}Ce_{x}O$ systems, from 1.39% to 12.50%. It is observed that by adding cerium to the zinc oxide system, the band of conduction was altered due to the *d* orbitals of cerium. As the cerium concentration decreased, the band gap also decreased; for this reason, the $Zn_{1-x}Ce_{x}O$ system could absorb visible light, improving its photocatalytic activity. The previous behavior was also reported in other theoretical and experimental studies [29,30,31,32].

On the other hand, in this study, we found that the systems $Zn_{1-x}Ce_{x}O$ did not manifest magnetization when x≥0.0278, while for lower cerium concentrations (x≤0.0185), a small magnetization occured for values close to 1.0 Bohr mag/cell.

**Figure 4:**
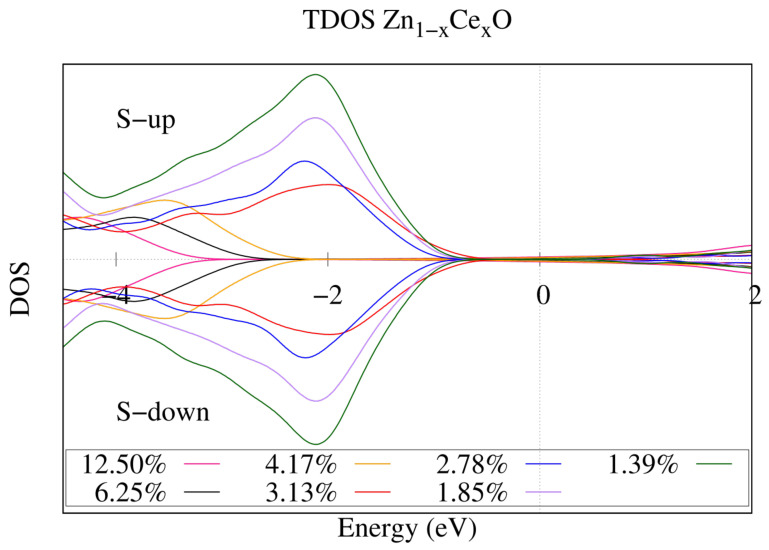
DOS for Cerium-doped zinc oxide at 12.50%; Cerium-doped zinc oxide at 6.25%; Cerium-doped zinc oxide at 4.17%; Cerium-doped zinc oxide at 3.13%; Cerium-doped zinc oxide at 2.78%; Cerium-doped zinc oxide at 1.85%; Cerium-doped zinc oxide at 1.39%.

Next, we aim to provide an explanation for the peaks observed in the photocatalytic activity. In general, the increase in photocatalytic activity of cerium-doped ZnO can be attributed to the decrease in the band gap, but it is not clear why the maximum photocatalytic activity rate occured at cerium concentrations close to 3.00% or 5.00%. To clarify the above, in Figure 4, we present the total density of states for the $Zn_{1-x}Ce_{x}O$ systems. It is observed that as the cerium concentration decreased, VB shifted towards the Fermi level; however, something interesting happened at a concentration close to 3.13%. It is seen that at this concentration, the maximum approximation of the VB to the Fermi level occured; therefore, there could be a relationship between the relative position of the Fermi level (closeness to the valence band) and the maximum photocatalytic activity observed for the system $Zn_{1-x}Ce_{x}O$ at a cerium concentration close to 3.00% [26]. This can be understood in this way, a closer proximity of the valence band to the Fermi level could represent more intermediate states (such as holes) in the band gap, which would allow for an increase in the recombination times of the electron–hole pairs photogenerated, resulting in a substantial improvement in photocatalytic activity.

According to the above, we have given an explanation for the peak in photocatalytic activity observed experimentally by [26] at a cerium concentration of 3.00%; however, our results we do not procide an explanation for the peak observed experimentally by [27] at a cerium concentration of 5.00%, so we believe that the highest photocatalytic activity for the $Zn_{1-x}Ce_{x}O$ system occurs for a cerium concentration of 3.00%

## 3. Materials and Methods

This study was implemented using Density Functional Theory (DFT) under the Quantum-Espresso computational package [34,35] because this package adequately reproduces the properties of periodic crystal systems such as those analyzed in this work, and it is widely used by the scientific community because it has shown reliable results. The Perdew–Burke–Ernzerhof (PBE) Generalized Gradient Approximation (GGA) [36] was used as an exchange-correlation functional (XC) with Vanderbilt ultrasoft atomic pseudopotentials [37] and a plane-wave basis set. The addition of the Hubbard term (DFT + U) with a U value of 7 eV for zinc was included. The atomic relaxation was carried out until the forces and energies were lower than $10^{-3}$ and $10^{-4}$ a.u., respectively, and the Broyden–Fletcher–Goldfarb–Shanno (BFGS) method was used [38].

**Figure 5:**
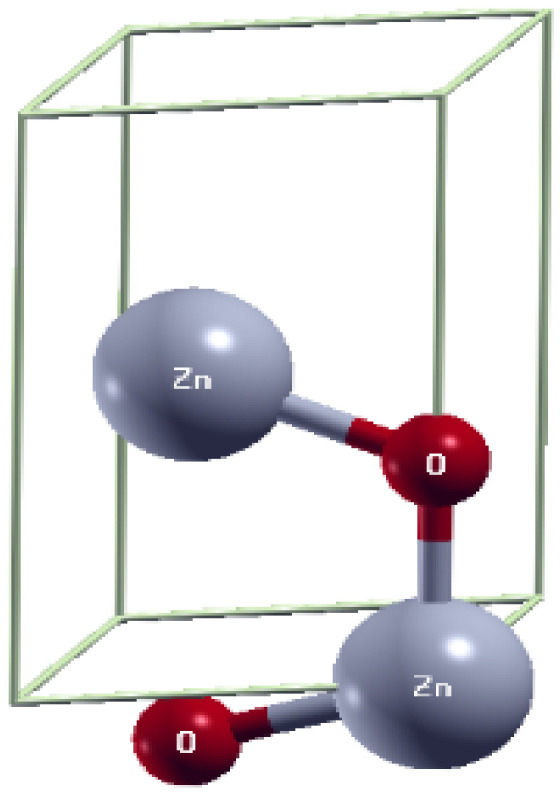
Unit-cell of zinc oxide in the wurtzite phase.

**Figure 6:**
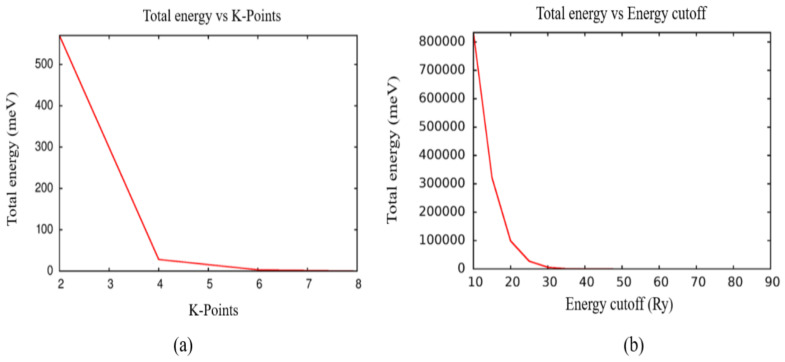
(**a**) Total energy as a function K-Points; (**b**) Study of the total energy as a function of cutoff energy.

The unit cell of zinc oxide bulk in the wurtzite phase (consisting of 4 atoms: 2 zinc and 2 oxygen) is shown in Figure 5. This allowed for the optimization of the input parameters as follows: To determine the k-point grid, a convergence study of the total energy was performed as a function of the k-points, as shown in Figure 6a; it is observed that there is a convergence from a value of 6, which corresponds to the grid of (6 × 6 × 3), which was used in this study. On the other hand, from a convergence study of the total energy as a function of the cutoff energy (as shown in Figure 6b), it was possible to establish an optimal value of 85 Ry and 850 Ry for the kinetic energy of the wavefunctions and the charge density, respectively.

After parameter optimization, Ce-doped hexagonal wurtzite ZnO supercells were created to study the influence of Ce doping on the structural properties, electronic properties, and photocatalytic activity of the ZnO system. Here, the oxygen atoms are red, zinc atoms are gray, and cerium atoms are purple.

**Figure 7:**
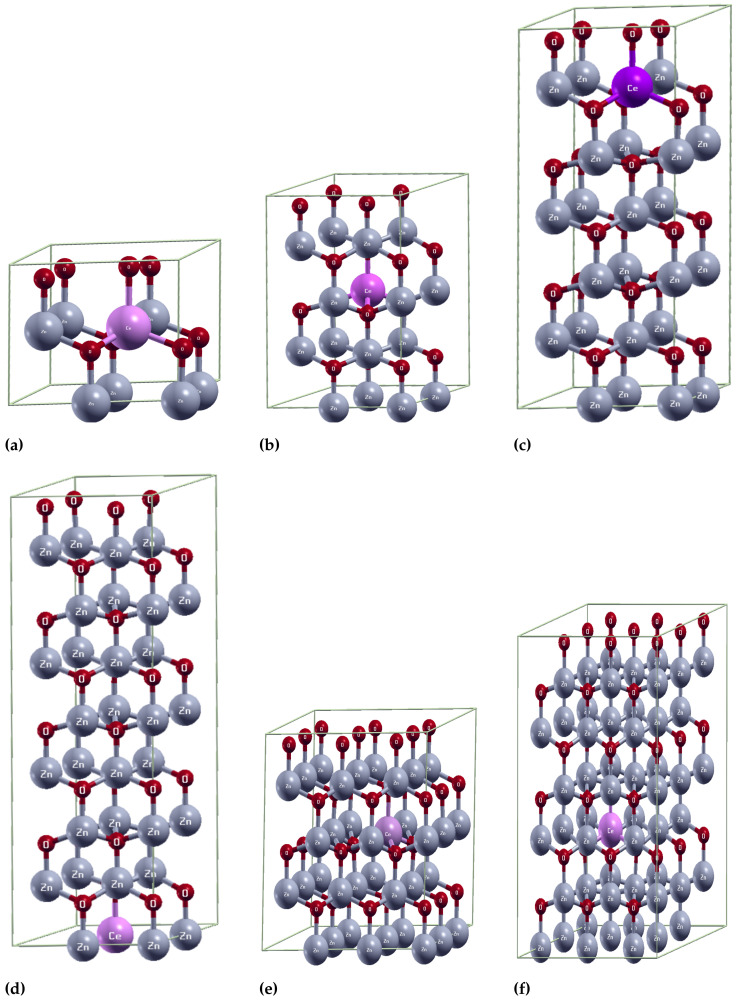
(**a**) Supercell 2 × 2 × 1/ $Zn_{0.875}Ce_{0.125}O$; (**b**) Supercell 2 × 2 × 2/ $Zn_{0.9375}Ce_{0.0625}O$; (**c**) Supercell 2 × 2 × 3/ $Zn_{0.9583}Ce_{0.0417}O$; (**d**) Supercell 2 × 2 × 4/ $Zn_{0.9687}Ce_{0.0313}O$; (**e**) Supercell 3 × 3 × 2/ $Zn_{0.9722}Ce_{0.0278}O$; (**f**) Supercell 3 × 3 × 3/ $Zn_{0.9815}Ce_{0.0185}O$.

To achieve a concentration of 12.50%, a 2 × 2 × 1 supercell was built, as shown in Figure 7a. It was formed by 16 atoms, of which 8 were oxygen, 7 were zinc, and 1 was cerium. Sequentially, the 2 × 2 × 2 supercell was created. It consisted of 32 atoms: 16 were oxygen, 15 were zinc, and 1 was cerium, as shown in Figure 7b. This allowed us to obtain a 6.25% cerium concentration. The 2 × 2 × 3 supercell allowed for obtaining a concentration of 4.17%. This supercell comprisesd 48 atoms, of which 24 were oxygen, 23 were zinc, and 1 was cerium, as shown in Figure 7c. For a cerium concentration of 3.13%, a 2 × 2 × 4 supercell was built, consisting of 64 atoms: 32 were oxygen, 31 were zinc, and 1 was cerium. This is shown in Figure 7d. The 3 × 3 × 2 supercell allowed for a concentration of 2.78%. This supercell comprised 72 atoms, of which 36 were oxygen, 35 were zinc, and one was cerium, as shown in Figure 7e. For a cerium concentration of 1.85%, a 3 × 3 × 3 supercell was built, consisting of 108 atoms: 54 were oxygen, 53 were zinc, and 1 was cerium. This is shown in Figure 7f. Although Figure 7 does not show it due to its similar behavior, a 4 × 3 × 3 supercell was also constructed, consisting of 144 atoms, of which 72 were oxygen, 71 were zinc, and 1 was cerium, which allowed for obtaining a 1.39% of cerium concentration.

## 4. Conclusions

In this investigation, the effect of cerium doping on the photocatalytic activity of zinc oxide was studied using Density Functional Theory (DFT). It was found that the highest photocatalytic activity rate for the $Zn_{1-x}Ce_{x}O$ system was associated with a cerium concentration of close to 3.00% and it could be due to the relative position of the Fermi level (closeness to the valence band). For a low cerium concentration in the $Zn_{1-x}Ce_{x}O$ system, the *c* lattice parameter had a lower value than that shown in pure ZnO, while the value of the *a* lattice parameter of the $Zn_{1-x}Ce_{x}O$ system was always higher than the value observed in pure ZnO. As the cerium concentration decreased, the band gap of the $Zn_{1-x}Ce_{x}O$ system also decreased, increasing its ability to absorb visible light. Additionally, it remains to be seen whether the relative position of the Fermi level in other systems plays the same role as that shown in this work in order to explain the peaks of photocatalytic activity for the $Zn_{1-x}Ce_{x}O$ system; if so, this methodology could be used to determine the concentrations of doping impurities for which the highest photocatalytic activity would occur in other materials. 

## Figures and Tables

**Figure 1:**
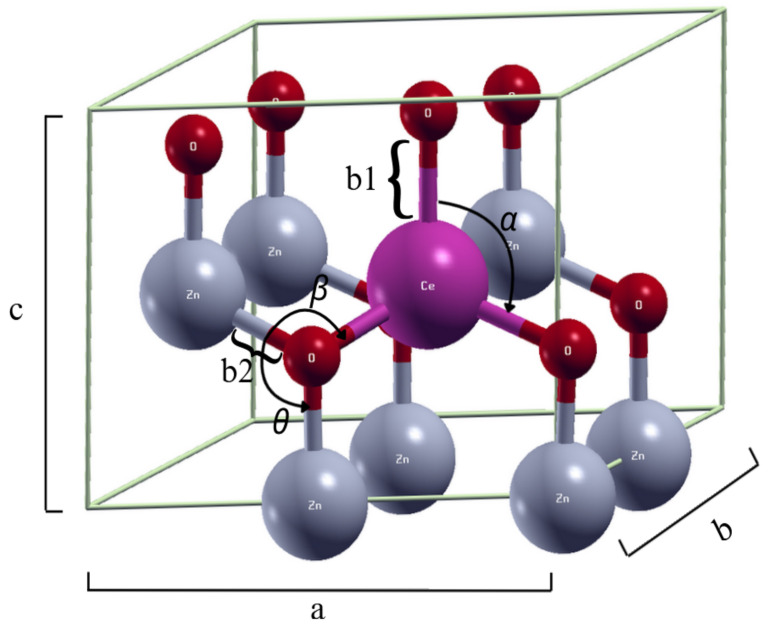
Representation of the bond lengths, angles, and lattice parameters (where a=b≠c).

## Data Availability

Data are contained within the article.
